# A commentary on the healthcare transition policy landscape for youth with disabilities or chronic health conditions, the need for an inclusive and equitable approach, and recommendations for change in Canada

**DOI:** 10.3389/fresc.2023.1305084

**Published:** 2023-12-14

**Authors:** Linda Nguyen, Claire Dawe-McCord, Michael Frost, Musa Arafeh, Kyle Chambers, Dana Arafeh, Kinga Pozniak, Donna Thomson, JoAnne Mosel, Roberta Cardoso, Barb Galuppi, Sonya Strohm, Alicia Via-Dufresne Ley, Caitlin Cassidy, Dayle McCauley, Shelley Doucet, Hana Alazem, Anne Fournier, Ariane Marelli, Jan Willem Gorter

**Affiliations:** ^1^ School of Physical and Occupational Therapy, Faculty of Medicine and Health Sciences, McGill University, Montreal, QC, Canada; ^2^ CanChild Centre for Childhood Disability Research, McMaster University, Hamilton, ON, Canada; ^3^ Cumming School of Medicine, University of Calgary, Calgary, AB, Canada; ^4^ Bachelor of Health Sciences Program, McMaster University, Hamilton, ON, Canada; ^5^ Patient and Family Advisory Council (young adult/patient partner), READYorNot™ Brain-Based Disabilities Project, CHILD-BRIGHT Network, Canada; ^6^ Patient and Family Advisory Council (Parent/Family Partner), READYorNot™ Brain-Based Disabilities Project, CHILD-BRIGHT Network, Canada; ^7^ McGill University Health Centre, Montreal, QC, Canada; ^8^ Schulich School of Medicine and Dentistry, Western University, London, ON, Canada; ^9^ Nursing and Health Sciences, University of New Brunswick, Saint John, NB, Canada; ^10^ Department of Pediatrics, Faculty of Medicine, University of Ottawa and Children’s Hospital of Eastern Ontario, Ottawa, ON, Canada; ^11^ CHU Mère-Enfant, Sainte Justine Hospital, Montreal, QC, Canada; ^12^ Department of Medicine, Faculty of Medicine, McGill University, Montreal, QC, Canada; ^13^ Department of Rehabilitation, Physical Therapy Science and Sports, UMC Utrecht Brain Center, University Medical Center Utrecht, Utrecht, Netherlands; ^14^ Centre of Excellence for Rehabilitation Medicine, University Medical Center Utrecht and De Hoogstraat Rehabilitation, Utrecht, Netherlands

**Keywords:** healthcare transition, equity, advocacy, human right, policy, disabilities, patient-oriented research, youth and families

## Abstract

There is a growing number of youth with healthcare needs such as disabilities or chronic health conditions who require lifelong care. In Canada, transfer to the adult healthcare system typically occurs at age 18 and is set by policy regardless of whether youth and their families are ready. When the transition to adult services is suboptimal, youth may experience detrimental gaps in healthcare resulting in increased visits to the emergency department and poor healthcare outcomes. Despite the critical need to support youth with disabilities and their families to transition to the adult healthcare system, there is limited legislation to ensure a successful transfer or to mandate transition preparation in Canada. This advocacy and policy planning work was conducted in partnership with the Patient and Family Advisory Council (PFAC) within the CHILD-BRIGHT READYorNot™ Brain-Based Disabilities (BBD) Project and the CHILD-BRIGHT Policy Hub. Together, we identified the need to synthesize and better understand existing policies about transition from pediatric to adult healthcare, and to recommend solutions to improve healthcare access and equity as Canadian youth with disabilities become adults. In this perspective paper, we will report on a dialogue with key informants and make recommendations for change in healthcare transition policies at the healthcare/community, provincial and/or territorial, and/or national levels.

## 1. Introduction

A growing number of children and youth with healthcare needs (YHCN) such as disabilities or chronic health conditions require life-long care (1–3). Psychosocial changes during the transition from adolescence to adulthood can be difficult for any individual. YHCN navigate the added challenge of transitioning from pediatric to adult healthcare services. The transition to adult services is defined as the “purposeful, planned movement of adolescents and young adults with chronic health conditions from child-centered to adult-oriented health care systems” (4). The most common age of transfer in Canada is 18, though it ranges from 16 to 25 years old for various services in different settings (5). The timing of transfer is set by policy, rather than by youth readiness. When the transition to adult services is suboptimal, youth may experience detrimental gaps in healthcare, increased visits to the emergency department, undue stress and poor health outcomes (6–9). Families have described the lack of preparation and access to adult healthcare services as “falling off a cliff” (10, 11).

Youth with complex healthcare needs are expected to transfer out of the pediatric system to access adult care services but require continuity of care (12, 13). Despite the rising number of YCHN entering adult services, there is limited legislation to govern the expectations of transition, including successful transfer, or to mandate transition standards in Canada. Current legislations about transition, including the age of transfer, vary greatly between provinces and territories (14, 15). Existing documents about transition are only guidelines or recommendations, which do not formally mandate adherence in practice. With varying implementation of guidelines and recommendations across Canada, transition preparation and follow through for YHCN depends greatly on the unique characteristics of the care environment in which they are receiving care, leading to inequities in access to supports and services.

There is an increasing need to support and empower youth during healthcare transition. In Canada, the federal government sets general health standards under the Canada Health Act and provides financial support for healthcare services to the provinces and territories. The provinces and territories are then responsible for administering and delivering health services, including the planning and funding of health facilities and implementation of health initiatives (16). The autonomy afforded to individual provinces and territories in determining health programming and funding responsibility has led to differences in healthcare transition planning for youth with disabilities and their families. Despite advocacy efforts from youth, families, and healthcare providers to bring attention to this critical issue, there have been few legislative changes, which may indicate a need for clear evidence to guide policymakers in their decision making (17, 18).

A position statement with calls to action to improve healthcare transition was recently published in Canada (19). It included a call for increased collaboration between pediatric and adult healthcare providers, as well as a streamlined approach for youth with disabilities as they transition to accessing adult health services across levels of care and sectors. The position statement further highlighted the critical importance of policy changes to support positive, successful transitions; for example, the need for flexible age cut-offs to ensure youth with disabilities are developmentally ready for the transition to adult healthcare, and the need for better access to developmentally appropriate transition planning for youth and families. The Children's Healthcare Canada Transition Hub (5) aligns with this call by uniting family and healthcare partners across the entire continuum of care (i.e., pediatric and adult care), ensuring that transition work is conducted collaboratively and in a coordinated manner, with a firm focus on policy change.

Further, a group of American organizations representing a variety of stakeholders recently identified the transition to adulthood as a health system research priority for YHCN (20). They developed *The Blueprint for Change* as a result of these meetings, and identified four critical areas to address, including health equity, family and child well-being and quality of life, access to services, and financing of services (21). With substantial care gaps worldwide, transitions that are less than optimal, lead to increased stress and vulnerability for YHCN. The inconsistency of planned, purposeful movement from pediatric to adult services amounts to a global health crisis for YHCN (22, 23). These recent examples illustrate the critical importance of addressing transition to adult care in a meaningful, consistent way, across populations and geographic areas.

Since healthcare transition challenges are not condition-specific, in this paper we take a non-categorical approach to the healthcare transition of youth with disabilities and a variety of healthcare needs, allowing national advocacy for change not only within, but also across conditions and families.

## 2. Dialogue with key informants

The CHILD-BRIGHT READYorNot^™^ Brain-Based Disabilities (BBD) Project (24) was initiated to develop and evaluate a patient-facing e-health intervention in four Canadian regions (Alberta, Ontario, Quebec, and the Maritimes). The MyREADY Transition™ BBD Application was designed to enhance healthcare transition readiness in youth with BBD. This project used a patient-oriented research approach to partner with a Patient and Family Advisory Council (PFAC) comprised of youth with disabilities and parents throughout all study phases (25, 26). PFAC meetings occurred regularly and included discussions about a range of topics related to healthcare transition, including how to advocate for changes in policy to improve healthcare transition experiences.

Based on these PFAC discussions, an advocacy working group was developed with the specific goal of identifying recommendations for policy changes in healthcare transition. Our working group collaborated with the Policy Hub (a rapid response unit for policy related to childhood disabilities) within the pan-Canadian CHILD-BRIGHT patient-oriented research network (27, 28).

A two-hour dialogue meeting was conducted in February 2022 with nine synchronous and one asynchronous participant from the four Canadian regions described above. The dialogue was co-facilitated and co-hosted by youth partners from the PFAC, with ethics approval from the Hamilton Integrated Research Ethics Board. Participants included three health care providers, one parent partner, four additional youth partners, and two researchers and/or healthcare administrators. To facilitate discussion at the dialogue, participants were presented with two patient vignettes and prompting questions (See Table 1). At the end of the dialogue, stakeholders were asked to identify their top three recommendations for policy changes. The dialogue was audio-recorded, transcribed, and the transcripts were analyzed using conventional content analysis (29). After reviewing the transcripts, themes were inductively identified; these themes are summarized below.

**Table 1:** Patient vignettes presented in dialogue.

Vignette 1
Patient name: Nadia Ayad Diagnoses: Epilepsy and generalized anxiety disorder Nadia, her younger sister, and her mom moved to Canada five years ago. When Nadia was in Grade 11, she collapsed during soccer practice and experienced a prolonged tonic-clonic seizure. She was then admitted to hospital for monitoring where she was diagnosed with epilepsy. During her stay, Nadia was frequently visited by Child Life specialists which helped her to feel less isolated. During her stay and after her discharge, Nadia and her younger sister often had to act as a translator between the doctors and their mom, who spoke little English. As a result, Nadia's mom didn’t have a full understanding of her daughter's condition and Nadia's sister, despite being younger, felt responsible for her care and this would often drive a wedge between the sisters. At school, Nadia feared that everyone would look at her differently if they knew she had epilepsy and so she tried to hide her condition. She quit her soccer team and became extremely anxious to leave the house for fear she might have another seizure and embarrass herself. School became a huge source of stress for her, and Nadia gradually began to isolate herself more and more. She was struggling to sleep at night and despite her sister's reminders, sometimes forgot to take her medication in the morning. That month Nadia had a seizure while writing a test in class and was sent back to the hospital. Nadia's mom wondered if her change in mood was the cause of her most recent seizure, but Nadia refused to ask that question to her doctor. It wasn’t until her sister brought up her recent anxiety and sleeplessness that her health care team set up an appointment for Nadia to see the school psychologist so that she could begin counselling for her anxiety. Counselling and medication greatly helped Nadia during the school year of Grade 11. She is now in Grade 12 and is planning to head off to university out of province this fall. Her family is worried about her transition and how Nadia will manage her conditions while in an unfamiliar environment. Nadia is excited to go, but she is already feeling overwhelmed by the amount of paperwork involved with university applications and disability support. When she was in high school, she didn’t have to worry about paying for counselling or applying for academic accommodations but now she is faced with having to complete many technical forms with little support from her mother.
Vignette 2
Patient name: Taylor Slessor Diagnosis: Autism spectrum disorder, asthma, cerebral palsy Taylor is an only child who lives at home with their parents and therapy dog in a house that was built by Taylor's dad to accommodate their wheelchair. Taylor was born premature and was diagnosed with cerebral palsy shortly after. Taylor's parents have been extremely involved in their care from day one, often speaking for Taylor when Taylor couldn’t. By the time they turned four, Taylor had also been diagnosed with autism spectrum disorder. Now in high school, Taylor currently receives most of their treatments in a children's rehabilitation centre. Before the COVID-19 pandemic, Taylor was attending a group life skills program to help them through their transition to adulthood. Since the pandemic, the program has been cancelled and Taylor has been isolating themselves and has been “acting out” more. Taylor was hoping to attend University in a city two hours away, this fall but given their recent challenges, Taylor's parents are questioning whether this is the right decision and are worried about campus accessibility, both from a physical as well as a sensory perspective. In a recent appointment with their family physician, the doctor raised the point that Taylor had never really been in charge of their own care, instead they relied primarily on their mother. Taylor stated that it was just easier that way and seemed uninterested in having to take control of their care moving forward. Taylor's family doctor made a referral to the campus health care team, but they said they were not equipped to handle Taylor's care and that Taylor would have to attend specialist appointments off campus.

## 3. What are the key elements of transition?

### 3.1. Transition taking place as a gradual process

Youth participants highlighted the importance of not having transfer “sprung on [them] at the last minute”, which can lead to additional stress. They advocated for a gradual process to prepare for transition, in which small goals can be reached to develop their confidence and skills to manage their health before transfer occurs. A researcher summarized the discussion shared among youth:

“It’s really about all of those small manageable goals so that if something’s not so hard and you can achieve it, it’s easier to imagine yourself making that next step or doing that next thing than if something is too big of a goal … it is about an ease of adaptability.”

Healthcare providers shared similar sentiments and highlighted the importance of planning early with youth and their families to set and achieve these goals before the transfer to the adult healthcare system occurs.

### 3.2. Provision of diverse resources and services for holistic care

Multiple youth shared that transition was more than just healthcare, and that transition to adulthood also includes education, social factors, finances, and transportation supports. One youth shared that he was not aware of financial disability support available to him until two years after he transferred to adult services. Youth also identified the importance of peer support and community resources as transition can often be a very lonely experience. One youth shared how a list of resources can be helpful to prepare for transition:

“I feel that if you give patients very early on a list of resources and groups they can join, or tell them that there are others in the community or other things, activities, and support groups in the community that they can join … I think that would have helped the whole process.”

Another youth described the importance of having resources and opportunities to practice skills that they would apply in adult care:

“It’s also important to not just give the resources but also know how to use them. Rather than giving a phone number and just saying, “Call this number.” To actually practice calling that number and what that looks like.”

Healthcare providers recognized the silo approach that often takes place in services when youth are transitioning to adult care where there is a lack of communication and disconnect between services. They expressed that the delivery of services needs to be changed.

## 4. What supports are needed for healthcare transition in practice?

### 4.1. Lack of training and resources for healthcare providers to support transition

Healthcare providers shared that there was not enough time for them to support their patients and families during transition, as transitional care was often a “side of the desk project”. A healthcare provider thought that:

“The people that work in transition do it because they have a passion and it’s not necessarily part of my paid role, but we fit it in because [we] believe that it’s important for our families, our children.”

One healthcare provider shared that she felt underprepared to transition her patients and that she learned everything on the job:

“At first, I was transferring my patients by making a good chart summary and telling them you're going to see this doctor over there, but my patients came back to me and said … “I was not ready”. So, I was really doing things wrong. So, I have to learn, with time, to do things differently.”

Overall, healthcare providers often felt that they had been undereducated on the complexities of preparing youth for transition. Based on their experiences, transition planning was only prioritized when a provider was willing to invest their own time and resources. They also stressed the importance of working as a team during transition planning, including patients and families, as well as multidisciplinary providers.

### 4.2. Inconsistent practices and lack of adherence

Participants further reinforced that transition policies and practices vary greatly from region to region, and even from provider to provider within a region. This uneven implementation of transition policies and practices can lead to inequities in access to services by youth and their families. One healthcare provider stressed the importance not just of having a policy but of also implementing and evaluating the policy:

“So, even if you have policies, and this is kind of a policy to say that this hospital has to have a transition program, otherwise, they don't get accredited, but then you have to look at **how they do it**. … so, it is not only the policy, and then put this policy in place … but then you **have to check** if it’s done well.”

Another healthcare provider described the importance of conducting research and evaluating transition outcomes related to implementation:

“Has this implementation really increased or improved any form of transitional care? And if it hasn't, that’s where you improve policy on a long term.”

Providers shared some examples of programs, in which they have participated and felt they were successful. However, they cautioned that the programs' success was often due to the involvement of a single “champion” provider, and it was important to consider the sustainability of these programs. One provider shared her dream of a Transition Bureau in each province and territory, which would have oversight of all healthcare transitions. These Transition Bureaus can communicate with each other to ensure that youth and families have access to the resources they require for healthcare transition.

Overall, both youth and healthcare providers felt that organizations should have clear local and regional policies for their practices with checks and balances when the transfer to the adult care system takes place.

## 5. Discussion

The themes discussed during the dialogue are consistent with published literature over recent decades (30–33), and informed the three recommendations proposed by the discussion group to help to prioritize advocacy initiatives and operationalize change. Transition requires collective responsibility from healthcare providers and provincial/territorial/national government systems. Table 2 summarizes recommendations for action, based on published literature (34) and our perspectives.

**Table 2:** Recommendations and collective responsibilities for healthcare providers, and provincial, territorial, and national government systems in Canada to improve healthcare transition.

Recommendations to advocate for change in healthcare transition policy	Actions		
	Healthcare providers	Provincial and territorial systems	National system
1. Flexible age of transfer (chronological age does not necessarily relate to developmental age or transition readiness).	Develop a local and/or regional policy with pediatric and adult health care providers for seamless transfer allowing for some flexibility.	Provide flexibility for the age of transfer for youth and their family who need it, which may include policy changes regarding eligibility for pediatric services and funding.	Develop national standards to assess readiness to transition, in particular for youth and families with complex healthcare needs.
2. Holistic transition to adulthood that includes the consideration of health, social and educational domains.	Build capacity in pediatric and adult care providers for holistic care and management of adolescents and young adults with childhood-onset conditions, including education about available resources. Build capacity in youth and families to empower them and develop self-advocacy skills. Promote awareness in young people and their families to optimize access resources and supports (funding, housing, education, and employment) for inclusion at the community level.	Collaborations across sectors, such as health, education and the social domain, with services working together to develop improved supports and access to those supports (including options for financial and transportation support, integrated education, accessible work environments).	Development of a federal framework designed to support an inclusive and equitable approach to transition for youth and emerging adults with healthcare needs, their families and caregivers.
3. Transition programming that begins a few years before transfer and allows time to build competencies.	Initiate conversations early with families; raise awareness of transition issues, including the barriers and facilitators, and provide reassurance to families; discuss the importance of developing self-management skills and autonomy as developmentally appropriate.	Ensure access to appropriate services and mandate early initiation of transition programming to create opportunities for youth and families to build competencies.	Promote the evaluation of transition services and share this evaluation with the public to ensure transparency; provide funding for longitudinal studies providing evidence on the long-term outcomes of holistic transition programming.

### 5.1. Recommendation 1: flexible age of transfer

The first, and perhaps the strongest recommendation from the group, is the need for a more flexible age of transfer (rather than a strict transfer date based on age) since chronological age does not necessarily relate to developmental age or readiness. There are several examples globally of recommendations advocating for a more flexible age of transfer to adult healthcare. For example, the National Health Insurance in Taiwan allows for individuals to access health services from all specialties regardless of age (35). The American Academy of Pediatrics (AAP) policy statement on pediatric age limits argues that 18 or 21 are nothing more than arbitrary numbers to choose to stop pediatric services (36). The AAP policy further suggests that pediatricians may be best suited to provide ongoing care, particularly for youth with complex needs and longstanding relationships with pediatric providers. The policy statement discourages the use of arbitrary age limits on pediatric care, highlighting the uniqueness of each situation, with age being only one of many considerations. Other factors in the timing of transfer can and should include the opinion of the patient/family, the training, abilities, and interests of the providers, with the providers being responsible for balancing their own abilities and limitations with the needs of the patient (36). Tools to benchmark and measure readiness for transition may help understand associated health outcomes (36). The European Alliance of Associations for Rheumatology (EULAR) Pediatric Rheumatology European Society (PReS) developed recommendations for healthcare transition for youth with juvenile-onset rheumatic diseases, and argued that the timing of the transfer could be flexible until the health condition is stable and when the provider considers the youth to be ready for the transfer to adult care services (37). They recommend flexible strategies such as providing opportunities for youth to communicate with adult services prior to the transfer, or having shared clinics between pediatric and adult healthcare providers (37). Such strategies and policies can and should be adopted across Canadian provinces and territories to offer flexibility in the timing of the transfer to adult care. This may include having patients, families and providers working together to agree on the minimum set of requirements to transfer so that healthcare transition can be done equitably and appropriately without a firm age cut-off.

### 5.2. Recommendation 2: holistic transition

Participants advocated for holistic transition preparation that includes consideration of factors beyond the traditional medical sphere. The transition out of high school to employment, postsecondary education or other post-secondary placements, and the transition to adult disability financial support programs were particularly highlighted. Holistic transition has been a common theme in the published literature as well, with youth and families describing concerns around housing, employment, financial and legal security after transitioning out of pediatric systems (38). Pediatric providers need to consider holistic transition in establishing a plan of care for patients who are transitioning to adulthood. In some cases, these transitional issues only emerge after the transfer to adult care has already taken place, and as such, adult providers need to be prepared to address these areas as well. Unfortunately, many pediatric and adult care providers report a lack of training in this area, and feel unprepared to meet the non-medical needs of patients leaving the pediatric system (39, 40). At a systems level, collaborations can and ideally should occur across sectors, with pediatric and adult services working together to prepare youth and families for the transition to adult services—both medical and social (41).

### 5.3. Recommendation 3: gradual transitions

Thirdly, participants recommended mandated transition programming that begins a few years before transfer and allows time to build competencies. The need to prepare for transition early was frequently highlighted by participants, and also represents one of the core components to support a successful healthcare transition outlined in position statements by both the Canadian Pediatric Society and American Academy of Pediatrics (19, 42). Having these conversations at least one year prior to transition and ideally even earlier, will allow time for youth/families to identify their goals, and develop necessary skills and abilities to take charge of their health (34, 43, 44). Youth with the capacity to do so can work towards gradually becoming more autonomous, with a progressive shifting of responsibility from parents/providers to the patients themselves (34). This may include having youth speak for themselves more in appointments, or taking on more tasks related to their daily care (e.g., refilling their medications) (45). The tasks to work on during this early phase of transition should be uniquely tailored to each individual but can only be a focus of clinical care if efforts are made to uniformly offer transition programming well in advance of transfer to adult services.

This project employed patient vignettes to generate discussion and identify recommendations to improve healthcare transition, laying the groundwork for a more comprehensive discussion with policymakers and health economists (46). Future work may benefit from a broader policy development framework such as the Narrative Policy Framework to understand the role of narratives in the policy process at different levels including at the micro (individual), meso (groups/coalitions/organizations), and macro level (institutions/society) (47).

Youth, families, and healthcare professionals are continuously advocating for policy changes to improve the transition from pediatric to adult healthcare. However, advocating for oneself or family member takes immense amounts of time and energy, and adds burden for families (48–50). Healthcare is a human right (51), and from our perspective, the onus should not be on the most vulnerable to engage in advocacy in order to have their human rights met. A future direction is for policymakers to create appropriate supports for individuals throughout the life course and across sectors.

It is critical to mobilize support for the dignity, rights, and well-being of YHCN throughout the transition to adulthood in Canada and internationally. We hope that this paper authored by youth, parents/caregivers, and healthcare providers is a starting point to advocate for change by providing actionable recommendations to improve transition outcomes for youth and families.

## Data Availability

The original contributions presented in the study are included in the article/Supplementary Material, further inquiries can be directed to the corresponding author.
